# “Turning science into video” Scientific communication for and with vocational students – a pilot study

**DOI:** 10.1186/s40900-025-00790-4

**Published:** 2025-10-28

**Authors:** Helen Koechlin, Sonja Schüler, Sabina C. Heuss

**Affiliations:** 1 Department of Psychosomatics and Psychiatry, University Children’s Hospital, University of Zurich, Zurich, Switzerland; 2 Division of Child and Adolescent Health Psychology, Department of Psychology, University of Zurich, Zurich, Switzerland; 3 Children’s Research Centre, University Children’s Hospital Zurich, University of Zurich, Zurich, Switzerland; 4 School of Business, Health Communication, University of Applied Sciences and Arts Northwestern Switzerland (FHNW), Olten, Switzerland

**Keywords:** Science communication, Co-creations, Young adults, Qualitative research, Associations with research, Personal understanding of scientific processes

## Abstract

**Background:**

Dissemination, i.e., the communication of research results to a wider audience, is often not part of the research process, resulting in significant delays in implementing new scientific results in practice. Young adults are an especially difficult group to reach with traditional methods of research communication, i.e., scientific publications, conferences, or panel discussions. Hence, new formats are needed to engage young adults in a dialogue with scientists and research results. The goal of this study was threefold: (1) the involvement of the target group from the start of the research project to the end and to find a topic of interest to young adults; (2) to jointly define a video format to present this topic and scientific results related to it; and (3) to evaluate content and format of the video in groups of peers not involved in the project.

**Methods:**

A Focus Group was conducted with vocational students (i.e., adolescents and young adults) to define a scientific topic of interest and the exact dissemination format by means of participatory research. Qualitative analysis of the transcribed Focus Group was conducted using structured content analysis. Further, surveys before and after watching the video were conducted in classes of vocational students not involved in the Focus Group to assess their images of science, their interest in science, and their opinion on the format and content of the video.

**Results:**

The Focus Group decided on the topic of stress related to school and education, and determined a group discussion with students, a professor in educational science, and a moderator from the research team. The video was well-received by peers, but almost half of participants would not watch another video like this. 21% of respondents state that the video has changed their understanding of science.

**Conclusion:**

The co-creation process used in this study has resulted in a topic of interest for the targeted audience. However, while watching the video changed perception and understanding of science in some recipients, they also questioned the added value with regard to learning something new.

**Supplementary Information:**

The online version contains supplementary material available at 10.1186/s40900-025-00790-4.

## Abstract (plain language summary)

Research produces results that are relevant for society, but very often, these results are slow to reach the group who would need them. One important reason for this is that a large and growing number of people no longer consume traditional media outlets (such as newspapers or radio). This is especially true for adolescents and young adults. In this project, we set out to examine whether a co-creation research format could be helpful to communicate scientific results to adolescents and young adults attending vocational schools. Students were invited to choose both the topic of the research and the format of the research communication (e.g. video). To ensure relevance of the topic to the target group, we closely worked with vocational students to (1) find a topic of interest to them (2), determine the exact content of the video, and (3) evaluate how the video was received by their peers. Participants in our study decided that they were most interested in stress related to school, and that they would like to discuss their individual experiences with a scientific expert in this area. Hence, the video had the format of a group discussion. While the video was well-received by participants’ peers in school, most of them said they would not watch another video like that in the future, and that they did not want to share it on their Social Media accounts. Together, this indicates that while videos are an attractive format to communicate scientific results, the question remains how the transfer of knowledge among peers can be improved.

**Supplementary Information:**

The online version contains supplementary material available at 10.1186/s40900-025-00790-4.

## Background

Dissemination, i.e., the active communication of scientific findings to a wider audience, is a complex part of the research process, and it might well take years, if not decades, for results to reach the targeted audience [1]. Reasons for this are manifold, and include problems around paucity of funding for dissemination, and a lack of knowledge on successful and targeted dissemination [2]. Further, the corona pandemic clearly uncovered difficulties in the dialogue between science and the wider public, and specifically a lack of successful communication about the mechanisms of science. During times of crisis, uncertainty and the sheer volume of news, updates and opinions make people vulnerable to believing and sharing unverified information [3, 4]. The news disseminated in the overload of information on social networks often originates from obscure or undisclosed sources. In addition, many people lack basic science literacy, meaning they do not fully understand how scientific research works, how evidence is evaluated, or why recommendations of conduct (such as instructions regarding masks or vaccines) may be issued and/or may change [5]. Miller [6] defines science literacy as “the capacity to understand scientific concepts and processes required for personal decision making, participation in civic and cultural affairs, and economic productivity.” The definition makes clear that science literacy is not just knowledge of facts, but the ability to apply scientific thinking in everyday life and societal contexts. Furthermore, growing social skepticism concerning institutions, including governments, media, and even the scientific community can be deepened by political polarization, which often turns scientific issues into ideological debates, making it even harder to convey objective facts [7, 8].

Adding to the complexity of the undertaking, an increasing number of people cannot be reached by traditional media outlets such as newspapers, radio, or television [9]. Low news consumption has proved to have negative impacts on crisis risk perception (the lower the news consumption, the higher the individual risk perception [10]), on the interest in politics, and participation in the political process [11]. A national survey on the habits of news consumption of different age groups in Switzerland showed that the percentage of the so-called “news-deprived” (i.e., people who show a news consumption that is far below average) 43%, with an increasing trend over time [12]. The term “news-deprived” was coined by sociologist and communication scientist Niklas Luhmann [13] and describes a group of people characterized by a significantly low consumption of media outlets, low interest in politics, and limited participation in democratic processes. Specifically, lower education level and younger age seems to be associated with being “news-deprived”: those aged between 18 and 24 years use little radio, television, and press [14].

Traditionally, research results are mainly disseminated through scientific journals and congresses, thereby excluding (young) people outside of academia from receiving research results that might affect them. For example, advantages in biomedical research might affect diagnosis and treatment of diseases, but without proper dissemination, the information remains inaccessible to those affected by the disease. Adolescents and young adults in particular have practically no or only very limited access to scientific information without considerable time and financial investment, and in conjunction with the language of science, this represents an insurmountable barrier for many [15]. Due to these barriers to scientific knowledge, the call for increased inclusion of the public in the research process has become more urgent in the last decades [16], calling for an involvement of the target group early and actively throughout the research process. This approach helps ensure that the research questions reflect the actual concerns and interests of the target group, and that the findings are communicated in ways that are accessible and meaningful to them [17]. In this sense, participatory research acts as both a communication and engagement tool.

However, even with more interest in participatory research practices, adolescents and young adults are still mostly overlooked: A recent systematic review found that fewer than 1% of studies in health research that include adolescent participants actually reported on the involvement of the target group throughout the research process, including dissemination of research results [18]. This is despite the fact that studies show how entertaining formats of science education can work for adolescents and young adults [19]. These so-called “Scientainment” approaches use visuals, a vocabulary that is tailored to the target audience, and distribute the content via new media channels such as social media [20]. The term “Scientainment” refers to the blending of science and entertainment in media formats that aim to inform and educate while simultaneously engaging and amusing audiences. According to Weingart [21], “Scientainment” reflects a trend in science communication where scientific knowledge is presented in entertaining ways to attract attention and increase public interest, but sometimes at the cost of depth and / or accuracy. This hybrid concept is increasingly used to describe how scientific content is communicated through entertainment channels such as television shows, Social Media, podcasts, or films, especially to reach broader or less traditionally engaged audiences. This concept is particularly relevant in the digital age, where younger audiences encounter science primarily through informal formats rather than traditional educational channels.

According to Schäfer et al. [22], science communication can be defined as any exchange of messages focused on scientific knowledge or scientific work, both within and beyond institutionalized science. This includes internal communication between scientists or institutions—for example, exchanges at professional conferences, internal emails, or daily operations in universities. It also includes external science communication, which occurs when scientists or research institutions communicate science-related messages to external stakeholders, such as the general public. The goals of science communication may include knowledge objectives, opinion-shaping objectives, behavioral goals, and emotion-related aims. At the level of goal-oriented design dimensions, information-oriented and dialogue-oriented objectives are particularly prominent today. For instance, discussions about scientific topics or new scientific findings are to be held with non-scientific actors from society, business, politics, and culture, and these findings should be conveyed through “translation” into non-scientific contexts. Objectives include fostering science literacy in target groups, in terms of understanding science and acquiring subject-specific knowledge.

In the field of dissemination research, the push vs. pull principle explains how youth either have to be interested in research and autonomously search for keywords or articles (i.e., pull principle) or that the content is brought closer to them through channels they already use (i.e., push principle in the sense of Hermida et al. [23]. In the context of digital and Social Media, the push approach is defined as a passive exposure to information through content surfacing users’ feed [23]. This shift toward ambient or incidental news exposure suggests that successful communication must meet audiences where they are, rather than expecting them to seek out information independently [23]. When trying to reach young people who primarily consume content via Social Media and mobile platforms—the push model becomes a vital strategy for disseminating research [23]. Following this, and considering the research on news-deprived groups, a push approach should support the dissemination of research results to youth in a successful way.

## Methods

### Aim

In this project, we aimed to initiate a dialogue between vocational students and scientists through a multi-step procedure: (1) by co-creation research and finding a topic of interest to vocational students with a Focus Group; (2) by jointly defining a dissemination format to inform their peers about the topic; and (3) evaluate the content and format of the video in groups of vocational students not involved in the Focus Group.

### Study design

Based on the background information and the objectives, hypotheses were formulated that guided the authors in the collaborative research approach. The authors assumed that the interest of vocational students about scientific methods and topics can be increased by the collaborative and participatory research method such as discussions, Focus Groups and ownership. Furthermore, the authors assumed that the interest in scientific methods and topics could be increased by newer dissemination methods such as videos or sharing content on Social Media.

**Fig. 1:**
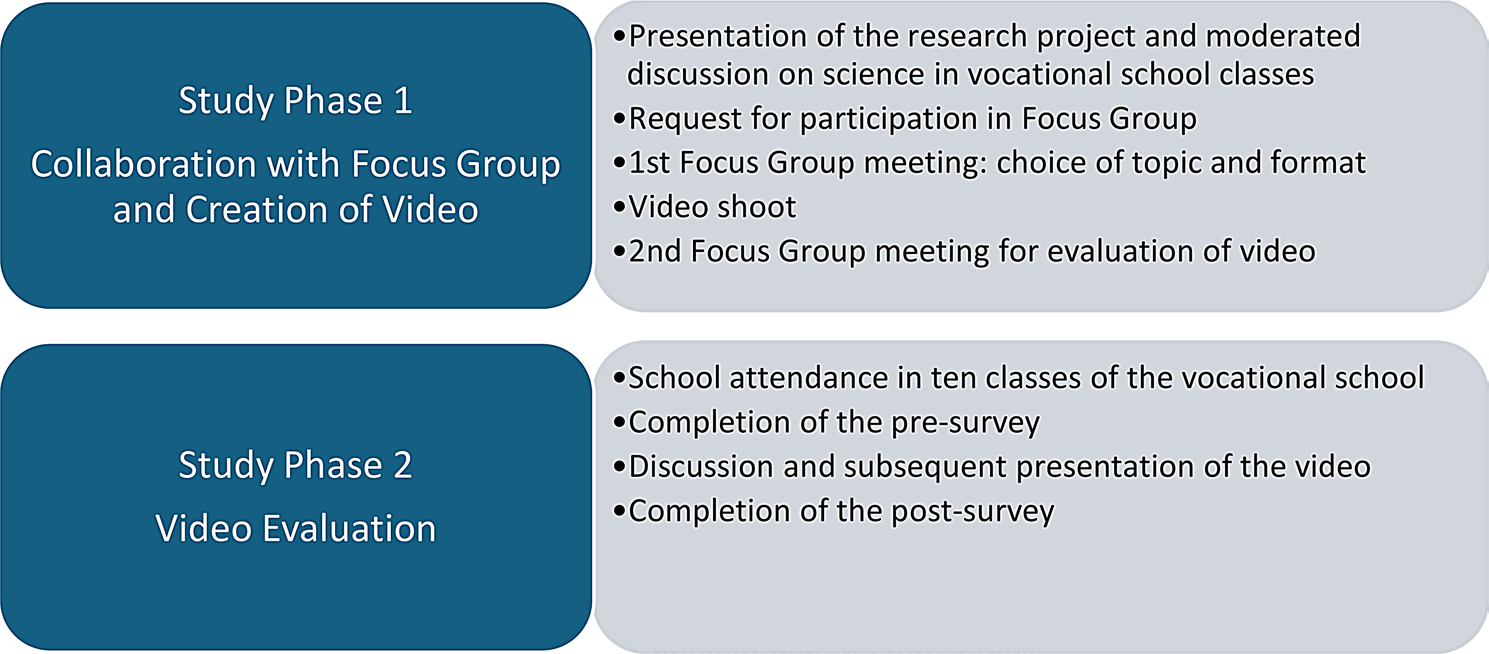
Study design

The study consisted of two phases: definition of scientific topic and video format through a Focus Group with vocational students (Study Phase 1), and evaluation of the topic and format by means of pre versus post surveys and discussions in vocational classes (Study Phase 2) (see Fig. 1).

A participatory and discursive approach was chosen to find suitable formats for science communication for the target group, to set the agenda for the video shoot, and to accompany the video shoot itself. The vocational students should be enabled to take the lead in finding topics and formats in a co-creation research form and to carry them out with the support of the researchers. Participation was always voluntary, and participants were reimbursed for their time with 150 Swiss Francs. Authorization from the ethics committee was waived by the Ethics Committee Northwest and Central Switzerland (Req-2023-00820) as the project was not defined as falling under Human Research Act (HFG Art. 2, Para. 1) [24].

### Participants

The study participants were students from a vocational school in the canton of Solothurn in Switzerland. The category of vocational students fits very well with the description of young people in Switzerland, who, according to the literature, are a larger group of news-deprived people and therefore less interested in research and science and were therefore chosen as a target group for this study. Since university students come into contact with research and science early on in their semesters, in this study we turned to a group of students who do not attend university but are nevertheless in a school setting and can therefore be easily involved and interviewed. In Switzerland, around two thirds of youth aged between 16 and 20 years attend vocational schools (i.e., they learn the skills required for a particular and specific job on the job and attend school for a few days a week) [25]. Hence, their educational environment provides a suitable place to disseminate research results.

Ten classes with between 8 and over 20 students, aged between 16 and 20 years old, took part in the final evaluation of the video. The vocational school students were participating in different vocational training programs and coming from different apprentice years in health, tailoring, animal care, hearing technicians, business administration and others.

### Focus group participants

In order to be included, participants needed to be students of one of the participating vocational classes and aged 16 years or above, agree to two Focus Group meetings, and potential participation in the video shooting. Five students aged between 16 and 20 years in their second year of vocational training in business administration, animal care and tailoring took part in the Focus Group. One of them identified as female, four as male.

### Procedure

In the first Study Phase (Study Phase 1), the project was presented to classes with different vocational training programs and apprenticeship years at a vocational school in Switzerland. This took place in January 2022. The authors presented the research project and initiated a moderated discussion with the students about their thoughts and stereotypes around science and scientists. Students were invited to participate in a Focus Group, a video shoot and a short online group discussion.

The work of the Focus Group began in March 2022 with the first meeting and ended in October 2024 with the second online meeting. The first meeting of the Focus Group with the five students was conducted in four sections (in the sense of Lüthje [26]): (a) introduction, presentation of the research project, the agenda, the discussion rules, and obtaining consent for the recording, (b) a warm-up with a conversation-stimulating initial stimulus, (c) the discussion with guiding questions, and (d) the conclusion and farewell. The Focus Group discussion was moderated by a member of the research team who focused on questions to determine the exact topic, and the format of the video to convey the content. The main aim of this phase was to enable the students to express their views on science dissemination. The aim was to select a common topic that was relevant to them and about which they would like to learn more from science. The students should also determine the form in which they would like to pass on their scientific findings to colleagues, or which format they would consider suitable for this. The moderator’s task here was to guide the discussion, but not to steer its content. A form of a co-creation research process should take place within the Focus Group, where the target group under investigation (the vocational students) can formulate their wishes, views and objectives independently with the help of the researcher (the moderator). The participants of the Focus Group were invited to take part in the video shoot and invited to a second online Focus Group meeting following the completion of the video to evaluate their experience.

The video was used as both intervention and assessment tools. As intervention tools, they helped simplify the rather complex scientific content, making it accessible, engaging, and emotionally resonant for the young audiences in the classrooms through the format of the group discussion between the five vocational trainees and the scientist. As assessment tool, the video was used to evaluate how audiences respond to science communication in the given format as such. The feedback of the vocational trainees allowed the researchers to measure shifts in knowledge, attitudes, trust, and behavioral intentions.

For the second Study Phase (Study Phase 2), i.e., the quantitative evaluation of the video, the video was uploaded onto a private, hidden page on YouTube and could be viewed, shared and commented on with the corresponding link. The research team visited ten vocational school classes in January 2024 to discuss the impact of the video with teachers and students. The students in the ten classes in the Study Phase 2 were first asked to complete the pre survey. The teachers then organized one lesson on science and research with PowerPoint presentations, inputs and discussions on the tasks and working methods of science and research and then showed the video. After the class discussion and watching the video, the students were asked to complete the post survey (see section on quantitative analysis). Vocational students were encouraged to share the video on Social Media, if they wanted to.

### Video

The video was shot in September 2023. The video documents a dialogue between a group of adolescents and a researcher, situated in the entrance area of a university building. Seated in a semicircle and equipped with handheld microphones, the adolescents engage in an open conversation about exam-related stress—a topic they selected themselves. Drawing on their personal experiences from school and vocational training contexts, they articulate specific stressors, triggers, and coping strategies. The researcher (a professor of school and teaching research from the Institute of Educational Science, University of Bern) responds by contextualizing their accounts within existing scientific evidence on examination stress and stress management. She highlights established findings from research and discusses their potential applicability in everyday educational settings. The video should serve a dual function: it should validate the adolescents’ lived experiences by situating them within broader societal patterns, and it should demonstrate how science can contribute to practical problem-solving. Moreover, it should emphasize the accessibility of scientific knowledge and fostered a sense of proximity between academic researchers and non-academic publics. The video, including intro and outro, was 10 min 24 s long and was streamed on YouTube.

### Qualitative analysis

The transcripts of the Focus Group meetings were analyzed using structured content analysis [27, 28], a multi-stage procedure for the category-based evaluation and hermeneutic interpretation of data, supported by the MAXQDA software package [29].

First, the dataset was transcribed verbatim and thoroughly reviewed through repeated readings and discussions to foster familiarity and identify initial analytic ideas and questions (S.S.). In the coding phase, relevant data segments were systematically identified and labeled with analytically meaningful codes [30]. The initial round of coding was collaboratively discussed until consensus was reached regarding coding levels and the inclusion of segments (S.S., H.K., S.H.). A second coding round followed, involving multiple review cycles, refinement of existing codes, and creation of new ones. Final codes were again discussed jointly.

Subsequently, codes were clustered into themes that captured broader, shared meanings within the data [30, 31]. These themes were further developed and reviewed by organizing them into thematic maps and evaluating their alignment with the full dataset and the overarching research question. The refinement process included ensuring internal coherence, consistency, and distinction among themes. In the final phase, each theme was clearly defined and named. The write-up aimed to address the research question by integrating analytic narrative and illustrative data extracts in a coherent and structured manner.

### Quantitative analysis

A standardized, two-part survey with open and closed questions (before and after watching the videos in class) was distributed to vocational students not involved in the research process. The survey was developed based on relevant literature in the field of science communication, especially based on the work of Schäfer and colleagues [15, 22, 32, 33] ensuring that key conceptual dimensions such as trust, interest, and understanding were addressed.

To support content and face validity, the survey was reviewed in several expert discussions between the authors and iteratively refined based on cognitive interviews with a small sample of participant interviews. These interviews helped ensure that the items were clearly understood and interpreted as intended.

The survey evaluated the images of science and researchers, students’ general interest in science, their views on the social role of science and scientific actors, their opinion of the video format, an eventual change in students’ opinion, attitudes and knowledge of science, research and their stakeholders, and whether this audiovisual format influenced the degree of their trust in science. All closed questions were rated on a 6-point Likert scale (see full survey in the Supplement).

We did not conduct a statistical reliability analysis such as Cronbach’s alpha, as the survey primarily comprised single-item measures rather than psychometric scales. While the instrument was not formally tested for internal consistency, several items were included in both the pre- and post-surveys, allowing us to track changes in participants’ responses over time.

The survey was conducted via the Tivian survey platform [34] and subsequently evaluated and analyzed with the statistical software R [35]. The responses to the open questions of the survey were manually analyzed using the same set of evaluation categories that had been developed in the context of the qualitative group discussion. This allowed the results to be meaningfully compared with findings from the other stages of data collection. The non-standardized elements of the quantitative survey were manually evaluated, processed under the evaluation categories of the qualitative data, and contrasted with the results of the other data collections.

## Results

### Study phase 1: results from discussing science with vocational students in class and from first focus group meeting

Three topics were identified from the discussion in vocational classes where the research project was presented and science and research were discussed with vocational students. These topics were also identified during the first Focus Group meeting: (1) Associations with research and science, (2) Reception of media coverage of science, and (3) Personal understanding of scientific roles and processes. These topics and all the statements below were collected prior to the video shoot.

Topic 1: Associations with research and science.

Question: “What comes to your mind when you think of science or research?”

The image of science among vocational school students was mainly influenced by natural sciences and technology when it comes to the disciplines associated with the terms “science” and “research”. The students in the Focus Group discussed that this might be because innovations in these disciplines quickly have a direct influence on people’s lives, that these disciplines are therefore disproportionately represented in the media – and that these are areas in which research often takes place at the interface between publicly financed science and the private sector.

The most described image of a scientist was a gray-haired, test-tube-wielding male and the figure of the modern, visionary science entrepreneur (Elon Musk was often used as an example). The following two statements by students from the classroom discussions are examples of the image they have of science as a whole or of individuals as representatives of scientists:

*“Elon Musk with his artificial intelligence and robotics… The things that you really do get to hear about… But certainly*,* also all the vaccines and medicines that are being distributed…”* Apprentice, 17 years old, hearing aid acoustician.

Medication, also in view of the corona pandemic, also with the development of the corona vaccines… Pfizer, Moderna…. Apprentice, 18 years old, health specialist

Topic 2: Reception of media coverage of science.

Question: “Where and how do you find out about science or are informed about science?”

The apprentices are sensitized to tendentious/manipulative as well as commercial media reporting and to the possibilities of manipulative media-mediated presentation of scientific research results in favor of private interests (groups). Accordingly, they consciously reflect on their own media consumption behavior and classify various (online) channels according to their information content, the selectivity of their reporting and their perceived trustworthiness.

*I’m on Instagram every day. I also follow 20 Minuten [a free newspaper in Switzerland]*,* because you see popular news there*,* and TikTok is also an information channel and you can get knowledge from it*,* but not always reputable knowledge… You always have to weigh up whether something is fake. You could see very clearly where the war in Ukraine started. There were videos of the war that were years old*,* video material that had nothing to do with the current war. People then panic*,* are misinformed.* Apprentice, 18 years old, optician

*TikTok*,* YouTube and so on… There’s a lot of money behind it. With the popularity of posts*,* you also get money… And some people are only interested in that and not in added value for society.* Apprentice, 17 years old, industrial painter

Topic 3: Personal understanding of scientific roles and processes.

Questions: “What does it mean to investigate something scientifically? And what is a “good” scientist in your opinion?”

The students discussed in the classroom discussions before watching the video what defines “good” science (students’ own choice of words). All the following characteristics mentioned of the image of a “good” scientist refer to statements made by the participants. Participants are convinced that “good” science is trustworthy science. In the eyes of the participants, competent scientific actors must have high intellectual and knowledge-related abilities, and work in a rule-governed, comprehensible and knowledge-oriented manner. In addition to scientific skills per se, a “good” scientist should also be able to demonstrate motivation and work ethos: “good” scientists should show passion for their own work, strive to produce scientific results that serve the good of the general public, and place the results they have achieved at the service of society. A “good” scientist should be characterized by a fact- and truth-oriented work ethos.

The participants also discussed how research results must be both accessible and intelligible to non-expert audiences. It draws attention to the necessity of conveying not only the outcomes but also the processes of scientific work in a way that is transparent and easily understood. Doing so fosters public trust and ensures that research maintains relevance within society. In contrast, the absence of such clarity can lead to a disconnect between scientific activities and the broader public, potentially undermining accountability.

*Above all*,* it has to be informative*,* so that the larger society simply knows what is being done. And how it is being done there. Because otherwise it means that someone is doing something*,* but no one has a plan of what should come of it in the end… So that you explain what you are doing. And not just in technical jargon*,* but in such a way that everyone can understand it.* Apprentice, 17 years old, logistician

With regard to their own perception of the trustworthiness of science, both views of science as a socio-economic engine of development, a factor in improving the quality of life and lifespan, and a source of knowledge were mentioned, but also concerns about a lack of transparency, social dangers, and the realization of particular interests through (pseudo-) scientific activities and discourses.

*I definitely have a very good feeling when it comes to medication or vaccines. It can cure cancer*,* for example*,* and I think about this progress for humanity*,* but then again*,* with certain things… artificial intelligence… depending on what you associate with it*,* it can definitely seem a bit… almost frightening.* Apprentice, 16 years old, health specialist

When students in the first meeting of the Focus Group were asked about their personal interest in participating in research, the topics of education and specifically of challenges in the students’ own educational careers became the core topic of the Focus Group discussion. “Stress in education” was agreed upon as the topic of interest for the video production. Students shared their own experiences with stress in their daily lives, and wondered whether, and if so, how, research results could relate to this.

They decided that the format of the planned video should be one of a panel discussion, during which students would share their stressors in school and confront an education expert with it. Additionally, the Focus Group defined that the video should be shot in a place where research happens.

In a second Focus Group feedback meeting after the video shooting, all participants of the Focus Group felt like this was a positive experience. The format of science communication was perceived by them as participatory, open, dynamic, and oriented towards exchange and discourse. All Focus Group participants expressed an interest in participating again in the production of comparable science communication formats in the future.

### Study phase 2: results from the survey

Once the video was finished, the research team visited ten vocational school classes and watched the video with them. Students filled out a survey before and after watching the video.

The survey was completed by 146 respondents (in the “before” survey conducted before viewing the video) and 139 respondents (in the “after” survey conducted after viewing the video), and had response rates of 96% and 95.2%, respectively. The participants of the survey part of the study were aged between 14 and 19 and consisted of people in training occupations of hearing system acoustician, healthcare professional, logistician, optician, painter, retail trade, and animal caretaker in different years of apprenticeship.

### Interest in science and research

**Fig. 2:**
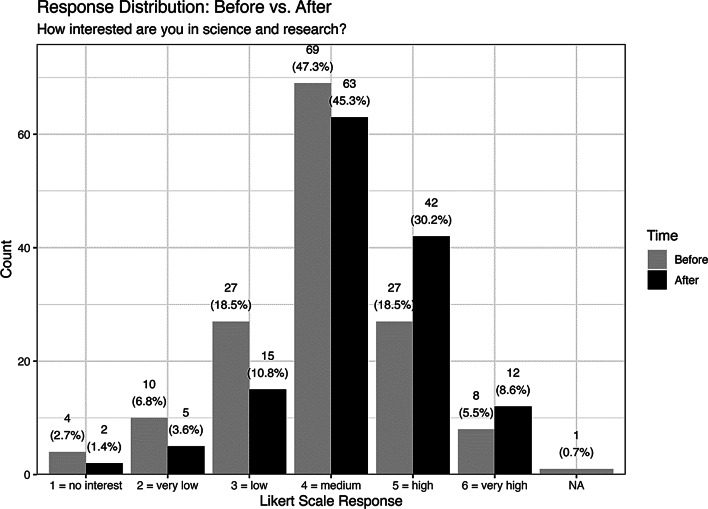
Distribution of responses before and after the intervention to the question: “How interested are you in science and research?” Responses are based on a 6-point Likert scale (1 = no interest, 6 = very high). A statistically significant shift toward lower (better) values was observed (Wilcoxon *p* = 0.0029), with a small effect size (Cliff’s delta = − 0.19, 95% CI: − 0.31 to − 0.07)

Before watching the Social Media video, roughly 25% of respondents reported a high or very high level of interest, while roughly 48% indicated a medium level of interest. In contrast, 28% expressed little to no interest in scientific topics. After the video and a guided discussion, students’ interest in science increased significantly. The share of respondents reporting a high or very high interest rose to 38.8%. At the same time, the proportion of those with little to no interest dropped to 15.8% (see Fig. 2).

### Trust

**Fig. 3:**
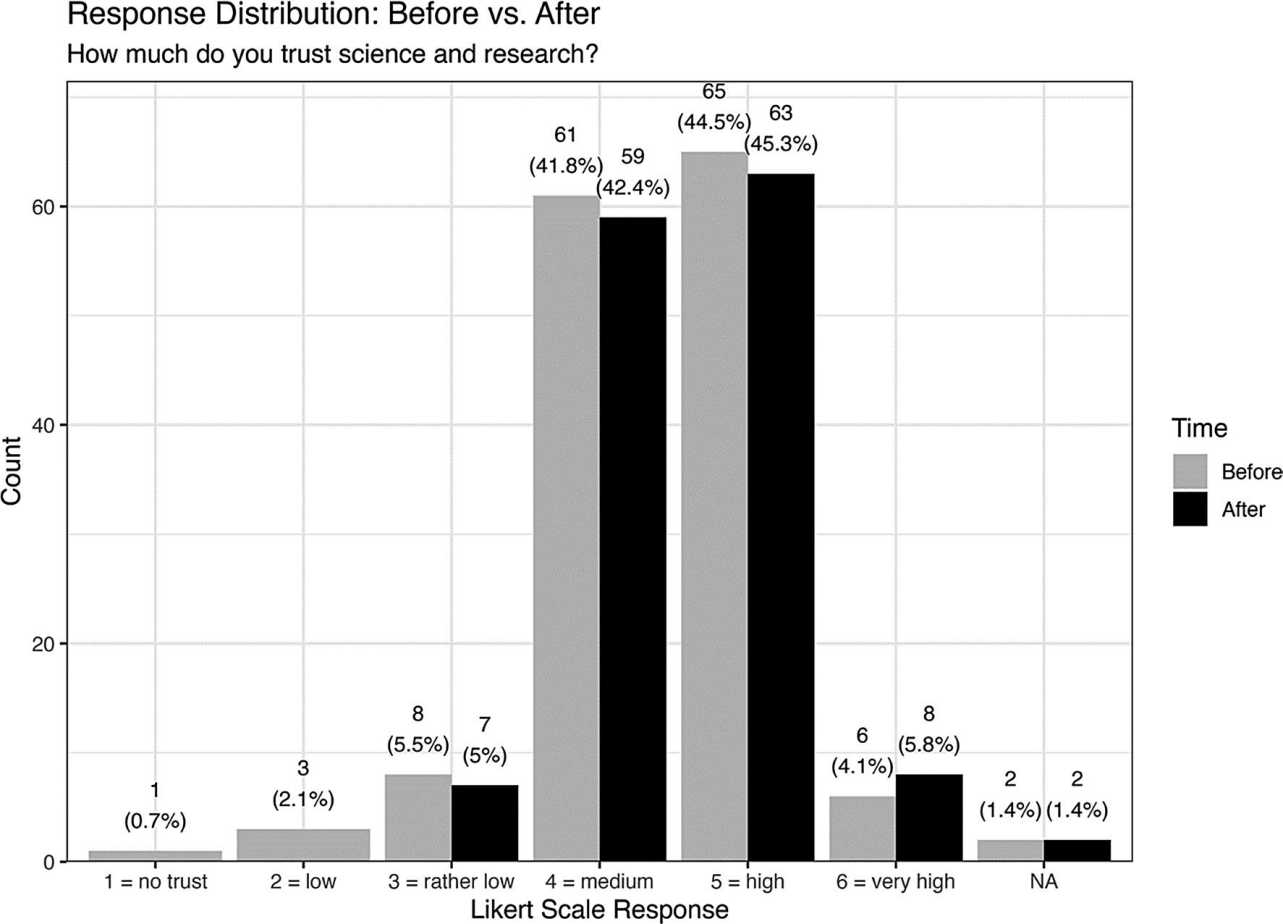
Distribution of responses before and after the intervention to the question: “How much do you trust science and research?” Responses are based on a 6-point Likert scale (1 = no trust, 6 = very high,). No statistically significant change was observed (Wilcoxon *p* = 0.45), and the effect size was negligible (Cliff’s delta = 0.05, 95% CI: − 0.08 to 0.17)

In the survey, we defined trust in science as the conviction that scientific methods, results, players, and institutions are reliable, credible and honest. The students’ level of trust was marginally changed by the video (see Fig. 3). At the end of the lesson on science and research and after viewing the video, 52% of respondents attested to a high or very high level of trust in science and research, which corresponds to a before-and-after increase of 3%. The proportion of students with little to no trust has fallen from 9% at the beginning to 4% after watching the video.

### Images and understanding of science

After watching the video, 21% of respondents agreed that their understanding of science changed. This was a closed yes/no question with the option to describe the change further. 17 participants provided additional description. Most of them indicated that science was much more than they expected. The proportion of those who changed their image of a scientist after watching the video was 17%. Some answers in the open question of the follow-up questionnaire as to what extent (if at all) the video had changed their own understanding of science reveal added knowledge regarding the scientific relevance related to the stress topic.

### Appeal of video

71% of the respondents stated that they liked the video, but almost half of them (49%) stated that they would not watch more videos in this format in the future.

### Reception of social media videos on YouTube

The Social Media videos on the University of Applied Sciences and Arts Northwestern Switzerland’s YouTube channel did not result in any significant discussion and exchange: During the five months of evaluation, the video “Practice meets theory: Exam Stress” was viewed 55 times. There were no likes or comments.

## Discussion

The present study developed and evaluated a video format to disseminate research results to vocational students, while involving the target group in the research process. Univocally, the students decided that stress around school and other duties was the most important topic for them, and that they would like to discuss their personal experiences with a scientist in this area. The formats of science communication have diversified greatly in recent years, particularly with regard to the challenges of reaching young target groups. The focus is increasingly on interactive, participatory approaches that are relevant to their lives [36], as also observable in our study. The discussions around the decision on the topic and the video shoot made science approachable and sparked interest through interaction at eye level, the greatest possible reference to the lives of the target group, mutual transfer of knowledge and interpretation, and a high degree of ownership, i.e. personal responsibility of the project participants.

Enthusiasm for participating in the project was tangible in all those involved and was reflected in the general commitment to further participation. Both the interest in science and research per se and the images of science and the science literacy of the vocational trainees turned out to be more pronounced than stipulated in the project hypothesis, even if this statement could only refer to topics that are familiar and important to them, such as exam stress and motivation. The comments of the vocational apprentices show a basic understanding of key scientific concepts and methods across disciplines such as biology, physics, and chemistry. They know how scientific knowledge is basically generated through observation, experimentation, and evidence-based reasoning. The comments show that they are able to distinguish between scientific facts and opinions. In the classroom discussions students critically assessed scientific information in media and understand the impact of science on society, and everyday life. Their comments also show an awareness of the ethical dimensions of scientific work.

A high degree of reflection regarding their own ideas about science, the ambivalent role of media in transferring scientific knowledge and the potential dangers of “fake science” under the aegis of particular interests became clear. In their trust in science and research, the apprentices are therefore cautious, and selective in their consumption of science-related content. This is in line with previous research exploring university students’ trust in information they encounter on Social Media that found a high awareness of participants regarding the existence of fake news [33]. What is more, others have described a surprising lack of trust, and a high level of skepticism towards the content on Social Media platforms [37]. In our study, we found that the innovative science communication formats of our video did not significantly increase trust in science and research - which was not to be expected from a single viewing of a video -, raising questions on how trust and the capability to distinguish between fake news and credible content can be improved: Trust is created through long-term interaction, transparency and personal relevance [38]. Hence, the effectiveness of formats such as ours in terms of building trust in science is still under debate.

All scientific activity must respect legal and ethical frameworks, values, and norms defined by society and politics. In this context, the concept of trust holds central importance: societal and political trust are key prerequisites for incorporating and defining the scope of scientific action and knowledge in political decision-making processes, public and civil society discourse, and everyday life [39, 40]. In order to ensure both scientific freedom and alignment with the common good, scientific actors depend on the interest and trust of the population as well as on exchange with the public.

A large study examining the population’s trust in scientists and their role in society across 68 countries found that most people trust scientists and consider them to be among the most trustworthy actors in society [41]. Nevertheless, there are differences in the extent of trust between different countries and population groups, as well as demographic differences: older people tend to trust scientists more than younger people, and people with a higher level of education often have comparatively more trust in scientific actors.

In addition, there is a difference between who to trust when it comes to science. Various studies come to the conclusion that distrust in science is often rather distrust in dominant governance, see for example [42–44]. This is supported by the work of Allchin [45] who explores the complexities of public trust in science and identifies the challenges faced by the general public in discerning credible scientific expertise. The study posits that while people generally trust science, they are often uncertain about which scientists or scientific entities to trust. This lack of clarity can undermine public confidence in scientific claims and lead to difficulties in engaging with scientific issues effectively.

Transparent science communication is crucial for building and maintaining mutual trust. Such participatory communication approaches aim to enhance acceptance, trust, and ultimately the willingness of non-scientific target groups to provide support. In some cases, actors in science communication link these knowledge- and behavior-related goals to the further aim of conducting research processes jointly with stakeholders from non-scientific contexts [46, 47], what exactly happened in the present research project. The dimensions of scientific research and innovation—such as content/results, actors, research questions, methodologies, benefits, and synergy potentials—must be communicated to stakeholder environments through target-oriented core messages. These messages should be conveyed in a tailored (visual) language via communication channels appropriate to the target group in order to achieve effectiveness in line with the defined communication goals [2].

These findings underscore the importance of factors related to life contexts for trust in science and, in line with the project results presented here, demonstrate the need to take these into account when designing science communication, thereby further emphasizing the need for participatory research and co-creation processes. A single, short-form video exposure appears insufficient to substantially influence trust. Furthermore, the dissemination and reception analysis revealed that the Social Media videos received minimal engagement, with very low view counts and no evidence of active interaction such as likes or comments. This outcome highlights a critical limitation of relying solely on passive dissemination strategies in science communication. As Joubert et al. [48] and others have argued, effective science communication increasingly depends on participatory formats that actively involve audiences rather than positioning them as mere recipients of information. Our study has shown: Active involvement was achieved through the Focus Group and class discussions in Study Phase 2, when the video was evaluated with the students.

Regardless of a certain level of reluctance of youth to trust Social Media content, audiovisual media, especially Social Media videos, offer a low-threshold way to spark interest in science [49]. In particular, YouTube, TikTok and Instagram have established themselves as central platforms for science communication as they create direct and informal access to science [50]. The results of the present study confirm this trend in part: participatory video creation and dialogue-oriented content design measurably increased *Focus Group participants’* interest in and reflection on science. Discussions in the vocational school classes showed that students use the aforementioned channels to access science-related content. However, a majority of students did not want to share the videos through their own Social Media channels despite encouragement, raising questions around how this peer-to-peer part of the dissemination process could be improved.

Our study in alignment with current research shows that students in vocational schools are often not sufficiently informed about the nature of expertise in specific scientific disciplines. The study calls for greater efforts in science communication that not only convey scientific knowledge but also elucidate the processes of scientific inquiry. The authors believe that trust in science can be reinforced, and the public can become more adept at navigating the complex landscape of science and research. The conclusion of the study emphasizes the importance of fostering open dialogue between scientists and the public and suggests that a more nuanced appreciation of who speaks and how science speaks is essential for promoting informed public discourse and engagement with scientific issues.

### Limitations

While this study has several strengths, some limitations must be acknowledged. First, the sample size and the specific educational context (vocational students within a Swiss institution) may limit the generalizability of the findings to other populations or educational systems. Cultural, institutional, and disciplinary differences can significantly shape responses to science communication interventions. Second, the intervention was based on a single short-form video, which inherently restricts the depth and breadth of engagement. The short exposure time and absence of repeated reinforcement likely limited the potential for measurable and lasting change. Third, the study relied primarily on self-reported measures collected immediately after the intervention. Although valuable for capturing immediate reactions, self-reports are susceptible to social desirability bias and may not accurately reflect enduring changes in attitudes or behaviors. Longitudinal follow-up assessments would be necessary to determine whether any observed reflections translated into sustainable effects over time.

## Conclusion and future directions

The results of our study advance research on science communication by showing that a co-creation process and an attractive video format can be effective in inspiring young adults to engage with scientific topics. At the same time, it highlights the need to understand science communication not only as a pure transfer of knowledge, but also as an interactive dialogue that promotes trust and critical thinking. While video-based science communication holds potential as an educational tool, its standalone use without interactive or participatory components appears insufficient to achieve meaningful and sustainable impacts on learners’ perception or trust in science. The passive nature of the peer-to-peer part of our dissemination strategy, especially the reliance on students to voluntarily share Social Media content, proved ineffective in stimulating meaningful digital engagement. This points to the need for more structured and potentially incentivized approaches when aiming to harness peer-to-peer communication channels. While the video format was appreciated by students and participants alike, it must be noted that this dissemination strategy is rather laborious, lengthy, and expensive, and might therefore not be suitable for routine scientific communication.

As engagement on Social Media platforms was low, therefore, questions around increasing engagement with science communication tools such as videos should be examined by future research – the duration of the video and humor could be two influential factors to explore further. Future research should tackle questions around improvement of peer-to-peer dissemination and explore ways on how to combine more passive formats (i.e., watching a video) with more active and interactive formats (such as panel discussions), which would also increase students’ ability to evaluate the credibility and trustworthiness of sources and information. More experimental formats that allow students to experience scientific phenomena in their everyday life, combined with digital tools and platforms to support curriculum might offer interesting avenues for future research.

## Supplementary Information

Below is the link to the electronic supplementary material.

**Supplementary Material 1:** GRIPP short reporting checklist

## Data Availability

Data and materials are available to other researchers upon request directed to the corresponding author. The Codebook is available upon request.
