# Supplementary material for: “Turning science into video” Scientific communication for and with vocational students – a pilot study
Source: Res Involv Engagem. 2025 Oct 28;11:128. doi: 10.1186/s40900-025-00790-4 (PMC12570461; doi:10.1186/s40900-025-00790-4)
Supplement: Supplementary file 1 — Supplementary Material 1: GRIPP short reporting checklist [file 40900_2025_790_MOESM1_ESM.pdf]

GRIPP short reporting checklist.

Section and topic	Item	Reported on page No
1: Aim	Report the aim of PPI in the study	3
2: Methods	Provide a clear description of the methods used for PPI in the study	3-6
3: Study results	Outcomes – Report the results of PPI in the study, including both positive and negative outcomes	6-9
4: Discussion and conclusions	Outcomes – Comment on the extent to which PPI influenced the study overall. Describe positive and negative effects	9-11
5: Reflections/critical perspective	Comment critically on the study, reflecting on the things that went well and those that did not, so others can learn from this experience	11
